# CaSR Induces Osteoclast Differentiation and Promotes Bone Metastasis in Lung Adenocarcinoma

**DOI:** 10.3389/fonc.2020.00305

**Published:** 2020-03-25

**Authors:** Lian Liu, Yichang Fan, Zhaoxin Chen, Yujian Zhang, Jing Yu

**Affiliations:** Cancer Center, Beijing Friendship Hospital, Capital Medical University, Beijing, China

**Keywords:** calcium-sensing receptor, proliferation, migration, bone metastasis, lung adenocarcinoma

## Abstract

**Objective:** Explore the mechanism of CaSR's involvement in bone metastasis in lung adenocarcinoma.

**Methods:** Immunohistochemistry (IHC) was used to detect the expression of calcium-sensing receptor (CaSR) in 120 cases of lung adenocarcinoma with bone metastasis. Stably transfected cell lines with CaSR overexpression and knockdown based on A549 cells were constructed. The expression of CaSR was verified by western blot and qPCR. The proliferation and migration abilities of A549 cells were tested using cholecystokinin-8 (CCK-8) and Transwell assays, respectively. Western blotting was used to detect the expression of matrix metalloproteinases MMP2, MMP9, CaSR, and NF-κB. The supernatant from each cell culture group was collected as a conditional co-culture solution to study the induction of osteoclast precursor cells and osteoblasts. Western blot and qPCR were used to validate the expression of bone matrix degradation-related enzymes cathepsin K and hormone calcitonin receptor (CTR) and osteoblast-induced osteoclast maturation and differentiation enzyme receptor activator of nuclear factor-κB ligand (RANKL), macrophage colony-stimulating factor (M-CSF), osteoprotegerin (OPG), and PTHrP. Immunofluorescent staining was used to detect F-actin ring formation and osteocalcin expression. Western blot results for NF-κB expression identified a regulatory relationship between NF-κB and CaSR.

**Results:** CaSR expression in lung cancer tissues was significantly higher than that in adjacent and normal lung tissues. The expression of CaSR in lung cancer tissues with bone metastasis was higher than that in non-metastatic lung cancer tissues. The proliferation and migration ability of A549 cells increased significantly with overexpressed CaSR. The co-culture solution directly induced osteoclast precursor cells and the expression of bone matrix degradation-related enzymes significantly increased. Osteoblasts were significantly inhibited and osteoblast-induced osteoclast maturation and differentiation enzymes were significantly downregulated. It was found that the expression of NF-κB and PTHrP increased when CaSR was overexpressed. Osteoclast differentiation factor expression was also significantly increased, which directly induces osteoclast differentiation and maturation. These results were reversed when CaSR was knocked down.

**Conclusions:** CaSR can positively regulate NF-κB and PTHrP expression in A549 cells with a high metastatic potential, thereby promoting osteoclast differentiation and maturation, and facilitating the occurrence and development of bone metastasis in lung adenocarcinoma.

## Introduction

Tumor metastasis is the leading cause of death in cancer patients and bone tissue is one of its most prevalent sites (1). Approximately 30–40% of patients with advanced lung cancer have bone metastasis, which seriously affects patient quality of life and prognosis. Active prevention and treatment of bone metastasis in lung cancer is of great significance (2). Tumor bone metastasis is a complex process involving multiple factors that interact with osteoclasts (OCs) and osteoblasts (OBs) in the bone microenvironment, promoting bone destruction and tumor proliferation (3, 4).

Calcium-sensing receptor (CaSR) is a widely expressed G protein-coupled receptor that is critical for maintaining metabolic balance between bone and calcium content. Abnormal expression of CaSR is closely related to the occurrence, invasion, and metastasis of various tumors (5, 6). CaSR can promote bone metastasis in breast cancer, prostate, and renal cancers (7, 8). It also has a low or negative expression in normal lung tissues (9). Our previous study has found that stronger bone metastatic ability in lung adenocarcinoma cells resulted in a higher CaSR expression. It was hypothesized that CaSR may be involved in bone metastasis in lung adenocarcinoma.

Parathyroid hormone-related protein (PTHrP) is an OC differentiation factor that plays an important role in tumor metastasis (10). PTHrP is mainly regulated by its upstream gene CaSR (11). When CaSR is upregulated and activated on the surface of tumor cells, it can stimulate the release of PTHrP from tumor cells (3). CaSR promoter region contains a functional response element κB and CaSR activation may be related to nuclear factor-κB (NF-κB). NF-κB is a core molecule involved in the response to the tumor inflammatory microenvironment and plays a key role in the expression regulation of various genes, especially those involved in immune inflammation (12). Studies have shown that activation of the NF-κB pathway in prostate tumor cells can stimulate tumor cells to secrete OC differentiation factors such as PTHrP and promote tumor metastasis (6). In addition, another study found that expression of PTHrP was significantly increased in tumor tissues of patients with bone metastases and that expression of PTHrP was positively correlated with bone metastasis in lung cancer (13). PTHrP can also directly induce OC differentiation and maturation by promoting secretion of IL-8, IL-6, IL-11, and other OC differentiation factors in tumor cells (14).

The present study first confirmed CaSR expression in lung cancer, lung cancer bone metastasis, and bone metastasis tissues. A549-based CaSR overexpression and knockdown cell lines were constructed to observe cell proliferation and migration abilities. After overexpressing and knocking down CaSR, the supernatant from A549 cells co-cultured with macrophages was collected to study CaSR promotion or inhibition of OBs and OCs. Western blot and qPCR were used to detect the expression of OB-induced OC maturation and differentiation enzyme genes and expression of genes involved in invasion. ELISA confirmed the expression of factors related to OC differentiation. The present study provided preliminary evidence to support the mechanism of NF-κB/CaSR/PTHrP signaling pathway in tumor cells to induce OC differentiation and maturation and promote the occurrence and development of bone metastasis in lung adenocarcinoma.

## Methods and Materials

### Patient Specimens

One hundred and twenty formalin-fixed paraffin-embedded (FFPE) lung adenocarcinoma samples were collected from January 2017 to October 2018, including 43 adjacent non-tumor tissues samples. The evaluation criteria for primary cancer are in accordance with the AJCC staging criteria. Patients were followed up until their death or the study's end date (2018-10-27). The median follow-up duration was 44.23 months (between 1.30 and 59.23 months). None of the patients received chemotherapy or radiotherapy before surgery. Patients who are eligible for chemotherapy received three to four cycles of platinum-based adjuvant chemotherapy.

### Chemicals and Reagents

Adenocarcinomic human alveolar basal epithelial cells line (A549 cells) was purchased from Shanghai Cell Library of Chinese Academy of Sciences; 1640 medium and superior grade fetal bovine serum were purchased from Hyclone; trypsin (C0203-100 ml) and protein quantification kits were purchased from Beyotime Biotech; CaSR antibody (ab137408, 1:1,000) was purchased from Abcam; MMP-2 (ab97779, 1:1,000) and MMP-9 (ab38898, 1:1,000) antibodies were purchased from Abcam; GAPDH antibodies (sc-365062, 1:1,000) were purchased from Santa Cruz Biotech; electrophoresis buffer and transfer buffer were purchased from Bio-Rad Laboratories; and goat anti-rabbit IgG (ZB-2301) was purchased from ZS Bio. IL-8 (ab214030), IL-6 (ab100712), IL-11 (ab189569) ELISA kit was purchased from Abcam; BCA protein concentration detection kit (P0010S) was purchased from Beyotime Biotech, plasmid extraction kit, total RNA extraction kit, reverse transcription reagent kit and DNA purification kit were purchased from Tiangen Biotech. ECL chemiluminescence kit (WBKLS0500) was purchased from Millipore Corporation. Cholecystokinin-8 (CCK-8) reagent was purchased from Dojindo Lab (Shanghai). NC membranes were purchased from Millipore and qPCR reagents were purchased from Vazyme Biotech.

### Immunohistochemistry

Immunohistochemistry (IHC) was used to detect the expression of CaSR in paraffin sections. Paraffin sections were deparaffinized, hydrated, placed in EDTA (pH = 8), and antigen was retrieved at high temperature for 5 min, and 3% hydrogen peroxide was used to block endogenous peroxidase for 15 min. After being rinsed with PBS buffer, CaSR antibody was added (Santa Cruz Biotech, diluted at 1:100) and kept overnight at 4°C. It was stained with streptomycin avidin-peroxidase (SP) for 30 min followed by DAB color development and hematoxylin counterstaining. Five high-power fields were selected for each slice to evaluate the results. Two pathologists used double-blind method to observe. Based on the number of positive cells, the criteria are as follows: –, 0 ~ 5%; +, 6 ~ 25%; ++, 26 ~ 50%, +++, 51 ~ 100%. A positive rate of more than 25% is defined as high expression, and a positive rate of ≤25% is defined as low expression.

### Cell Culture and Establishment of a Stable CaSR-Overexpression Cell Line

Human A549 cells were cultured in a 1640 medium, and the plasmid carrying the CaSR gene and three helper plasmids were transferred into A549 cells by lentivirus infection, and the CaSR gene was inserted into the genome of the cells. GFP-positive cells were selected by flow cytometry. Monoclonal clones were selected to inoculate a 96-well plate. Fluorescence microscopy was performed within 2 weeks. Positive clones were transferred to a 6-well plate to expand culture and appropriate CaSR overexpressing stable cell lines were selected according to the expression level. At the same time, cell lines with empty plasmids were constructed, labeled oeCaSR and oeControl. Similarly, the hairpin structure and the nonsense shRNA sequence were designed according to the mRNA sequence of CaSR in GenBank, and the CaSR knockdown cell line and control cell line were constructed and labeled as siCaSR and siControl. The CaSR siRNA (h) sequence was purchased from Santa Cruz Biotech (Cat. No. sc-44373) and the Control siRNA (h) sequence was purchased from Santa Cruz Biotech (Cat. No. sc-37007).

### *In-vitro* System of Inflammatory Microenvironment and Preparation of Conditioned Medium (CM)

According to our previous study (15), we used THP-1 cells to be seeded on 6-well plates at 1 × 10^6^ cells/well. The THP-1 monocyte cells were converted to macrophages using Phorbol 12-Myristate 13 acetate (PMA) purchased from Sigma-Aldrich (Shanghai, China) at a final concentration of 320 nM and incubated at 37°C with 5% CO_2_ for 48 h to allow for maximal conversion of the THP-1 monocytes to THP-1 macrophages. All the experiments were performed in polystyrene with Transwell inserts with a pore size of 0.4 μm from BD Biosciences (San Diego, CA, USA) using serum free medium. The co-culture system consisted of adding an insert containing confluent THP-1 macrophages to cultured A549 cells that are grown in the bottom compartment of the plate. The THP-1 macrophages were never in direct contact with the A549 cells. In the co-culture system, the A549 cells with different CaSR expresssion were divided into four experimental groups, that is oeCaSR, oeControl, siCaSR, and siControl groups. According to the method of Ell et al. (16), the A549 cells and macrophages of each experimental group were co-cultured for 24 h, and the supernatant was collected, filtered through a 0.22 μm filter, and stored in a refrigerator at −20°C. The conditioned medium (CM) was collected and mixed in a sterile bottle to avoid deviations in the cell growth rate and the number of cells in each of the collected co-cultured cell culture supernatants. CM was then dispensed and stored in a refrigerator at −20°C for use.

### Cell Isolation and Identification

Differentiation and identification of osteoclast precursors: 20 mL of fresh peripheral blood of volunteers were aseptically extracted, and a single nuclear cell layer was obtained after centrifugation and inoculated into a cell culture flask or a culture plate. The media were α-MEM with 10% FBS, 100 U/mL penicillin, 100 μg/mL streptomycin, 35 ng/mL macrophage colony-stimulating factor (M-CSF) and 40 ng/mL murine recombinant receptor activator of nuclear factor-κB ligand (RANKL). After 12 days of culture, according to the TRAP staining method of Mostafa et al. (17) it was stained for 30 min followed by light microscopy and TRAP positive cell count. Cells with more than three nuclei and positive for TRAP staining were considered osteoclasts. TRAP^+^ multinucleated cells were counted under a 400-fold random-selected five fields of view. After 15 days of intervention of each group, five fields were randomly selected from each group under a 400-fold field of view, and the size of osteoclastic area was calculated as the percentage of the entire field of view. The area of TRAP-stained oesteoclasts was calculated with Image J software (NIH). In this study, the rat calvarial cells were isolated as osteoblast precursors and cultured using the Orriss method (18). The experimental cells used were all 2nd generation cells. The morphology of osteoblasts was observed by inverted phase contrast microscope. Cell mineralization nodule staining was performed with alizarin red S (AS). Pictures were taken under the microscope. The area of AS-stained oesteoblasts was also calculated with Image J software (NIH).

Osteoclastogenesis and osteogenesis assay were performed also in presence of CM from the aforementioned four groups of the co-culture system to examine the potential of inducing osteoclast and osteoblast differentiation.

### Tumor Cell Matrigel Invasion

After A549 cells were digested, the cells were seeded at a density of about 2,000 cells per well to a layer of Matrigel in the 8 μm pore size transwell chamber (354480, Corning, USA). One thousand six hundred forty with 1% FBS medium was added to the upper layer and 1,640 with 10% FBS was added to the lower layer. After 24 h of culture, the cells in the upper layer of the transwell chamber were gently wiped off and washed 3 times with PBS. The lower cells were fixed with 4% formaldehyde for 20 min, dried at room temperature for 30 min, and stained with 0.1% crystal violet stain for 30 min. After rinsed with deionized water, observe the staining results under a light microscope. Five high power fields were randomly selected for cell counting. After elution with 500 μl of acetic acid per well, the absorbance of the eluate at 570 nm was measured with a fluorescent plate reader and recorded.

### RT-PCR

A549 cells were cultured in 60 mm culture dishes. Total RNA was extracted with the Total RNA Isolation System. cDNA was synthesized from 3 μg total RNA with the Reverse Transcription kit. All qPCR was performed with a Roche Light Cyclers 480II Detection System with SYBR green incorporation according to the manufacturer's instructions and actin was used as an internal control. The primers were acquired from the published literature and the Primer Bank (http://pga.mgh.harvard.edu/primerbank/) and are listed in Table 1.

**Table 1:** The primers list in qPCR assay.

**Target gene**	**Sequence**	
Human Cathepsin K	F	5′-ACTGGACTCAAAGTACCCCT-3′
	R	5′-GCCATCATTCTCAGACACAC-3′
Human CTR	F	5′-ATTTGGCTATTGTGGGTCA-3′
	R	5′-CATGTTCTTGTGCAGGGT-3′
Human Cathepsin K	F	5′-ggcaaactcttagctctg-3′
	R	5′-ctctgtaccctctgcac-3′
Human CTR	F	5′-CTCTGGTGGTCAACTTCTTCT-3′
	R	5′-gggcctccagggaaacac-3′
Human GAPDH	F	5′-TATCGGACGCCTGGTTAC-3′
	R	5′-CTGTGCCGTTGAACTTGC-3′
Rat GAPDH	F	5′-CTCAAGATCATCAGCAATG-3′
	R	5′-GTCATGAGTCCTTCCACG-3′

### Western Blot

The cell culture medium was removed, washed twice with PBS and the cells were scraped off. Pre-chilled cell lysate was added, and the sample was lysed on ice for 30 min. Protein content was determined by BCA method. Twelve percentage 1D SDS-PAGE gel electrophoresis were carried out. The protein was transferred to a NC membrane. After blocking with 5% skim milk for 1 h at room temperature. The NC membrane was incubated with the corresponding primary antibody at 4°C overnight. And then, the membrane was washed with TBST for 20 min and it was incubated with the secondary antibody for 1 h at room temperature. The membrane was washed for 20 min with TBST and developed with ECL reagents (Engreen, China). GAPDH was used as an internal control and detected by the GAPDH antibody. Image J software (NIH) for image processing was used to verify the band intensities as a result of semi-quantitative western blot. The software generated peaks according to the band intensities and the expression was compared between samples and controls. The experiment was repeated three times.

### Enzyme-Linked Immunosorbent Assay (ELISA)

According to the kit operating instructions, in brief, a 96-well-plate was coated by the coating antibody overnight followed by blocking for 2 h with blocking buffer. Then CM of the four groups from the co-cultured modified A549 cells with macrophages were added on the plate and incubated for 2 h. After adding the detection antibody for 1 h incubation, the secondary antibody was added and incubated for 1 hour. The plate was washed four times with washing buffer after each step above. After 5 min incubation with substrate, the stop solution was added and the absorbance was read on a microwell plate reader at a wavelength of 450 nm within 10 min. The experiment was performed at room temperature and all the samples were assayed in duplicate.

### Flow Cytometry for Characterization of Oesteoclasts and Osteoblasts

Oesteoclasts were stained and characterized with PE conjugated mouse anti-human RANK (Invitrogen, cat. no. MA1-41015) using appropriate controls. Cells were incubated for 15 min on ice with CD16/32 (FcγIII/II receptor; 1:100; FcBlock™; Pharmingen) before staining with the first antibody. 10^6^ cells/100μl were suspended in 5% FCS/PBS (washing buffer). Cells were stained with the anti-RANK antibody, incubated for 30 min on ice, and washed twice with washing buffer. The secondary antibody was added, and the cells were incubated for 30 min on ice. After incubation, cells were washed twice with washing buffer.

Osteoblasts were trypsinized, washed with PBS and then fixed with 4% paraformaldehyde. Thereafter, cells were permeabilized using 1% BSA with 0.1% Triton X-100, blocked with 2% BSA. Intracytoplasmic staining of differentiated cells was done using PE conjugated Osteocalcin (R&D Systems, cat. no. IC1419V) using appropriate controls. Data was acquired using FACSCalibur (BD Biosciences, San Jose, CA, USA) and analyzed using FlowJo v7.6.1 software (Tree Star, Ashland, OR).

### Immunofluorescent Staining

To evaluate F-actin ring formation, osteoclasts cultured on bovine bone discs were fixed with 4% paraformaldehyde for 15 min at room temperature and permeabilized with 0.1% Triton X-100 for 5 min. The cells were then stained using the Acti-stain™ 488 phalloidin (Cytoskeleton Inc. PHDG1-A) at room temperature in the dark for 30 min. The formation of the actin ring was captured using the systems NISC Elements software and analyzed using ImageJ.

For osteocalcin immunofluorescent staining, osteoblasts were fixed with 4% paraformaldehyde for 15 min at room temperature and permeabilized with 0.1% Triton X-100 for 5 min. The cells were then stained using the osteocalcin (ab13418, 1:200) at room temperature in the dark for 30 min. The cells were washed with PBS, and the nuclei were then counterstained with 4',6-diamidino-2-phenylindole (DAPI, Sigma Aldrich; Merck KGaA). Fluorescence images were collected using the systems NISC Elements software and analyzed using ImageJ.

### Statistical Methods

Data analysis was performed using SPSS 17.0 statistical software. Measurement data were expressed as mean ± standard deviation (x ± s) and *t*-test was used for comparison between the two groups. Count data were expressed as rate and the comparison between groups was performed by χ2 test. Correlation analysis was performed using Spearman rank correlation test. Difference was statistically significant at *p* < 0.05.

## Results

### CaSR Is Highly Expressed in Lung Cancer, Lung Cancer Bone Metastasis, and Bone Metastasis Tissues

**Figure 1:**
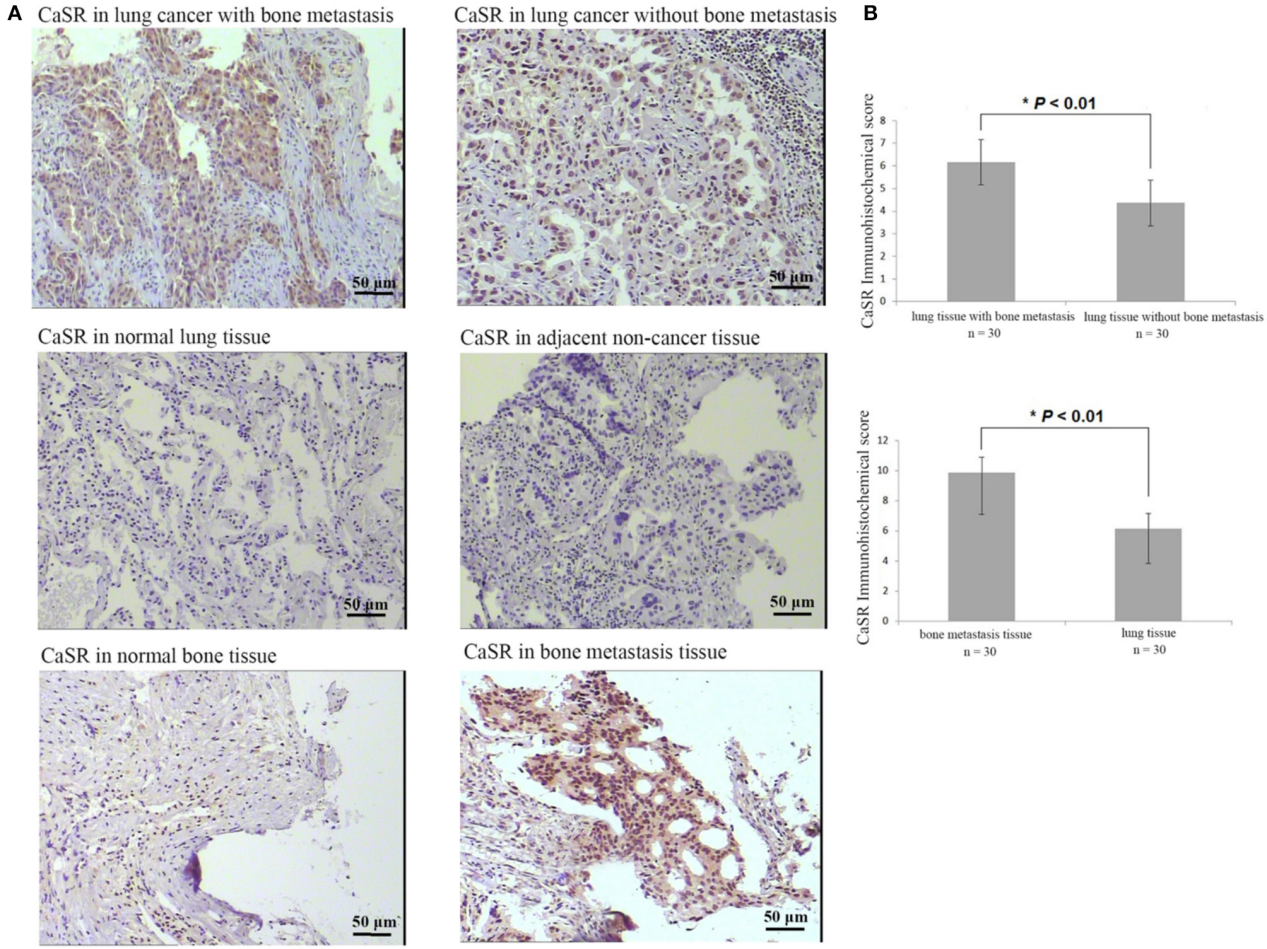
Analysis of CaSR expression in lung cancer tissues, lung cancer bone metastasis tissues, and bone tissue metastasis samples. **(A)** Typical immunohistochemistry (IHC) staining results. **(B)** In lung cancer tissues, CaSR expression was significantly higher in bone metastasis group than in non-bone metastasis group (*p* < 0.01). In bone tissues, CaSR expression was significantly higher in bone metastasis group than in normal group (*p* < 0.01). *p < 0.05.

Immunohistochemical analysis of 120 patients with lung adenocarcinoma demonstrated that CaSR expression was significantly increased in lung cancer tissues of lung adenocarcinoma patients with bone metastases compared to patients without. CaSR expression in bone metastasis patients was significantly higher than that in primary lung cancer tissues (*p* < 0.01). Its expression in normal lung tissues was low or negative. The typical case staining results are shown in Figure 1, suggesting that CasR was activated in lung cancer bone metastasis and may be involved in the process of bone metastasis.

### Construction of CaSR Knockdown and Overexpression Cell Lines

**Figure 2:**
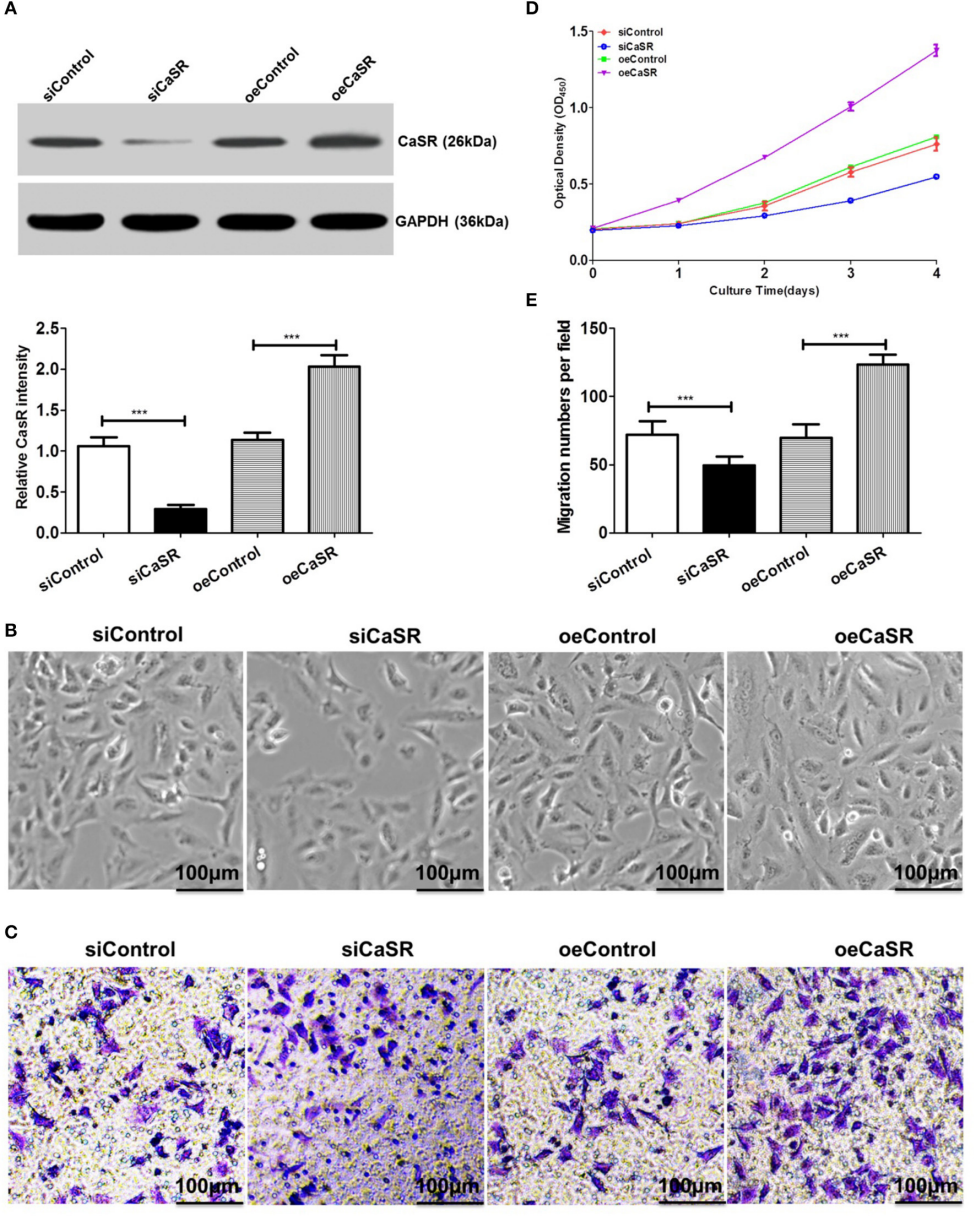
Analysis of proliferation and migration abilities in A549 cells after CaSR knockdown and overexpression. **(A)** A western blot was used to verify CaSR expression level after cell line construction. CaSR protein expression level in A549 cells was significantly higher in CaSR overexpression group than in control group. CaSR protein expression level in A549 cells after RNA interference was significantly lower than that in negative control group (by about a factor of 0.08; *p* < 0.001). **(B,D)** CCK-8 was used to detect A549 cell proliferation. A549 cell proliferation ability was significantly higher in CaSR overexpression group than in control group. A549 cell proliferation ability was significantly lower in CaSR knockdown group than in negative control group (*p* < 0.001). **(C,E)** A549 cell invasion ability was examined by transwell assay. Number of transmembrane cells was significantly greater in CaSR overexpression group than in control group and the difference was statistically significant (*p* < 0.001). Number of transmembrane cells was significantly lower in CaSR knockdown group than in negative control group and the difference was statistically significant (*p* < 0.001). ***p < 0.001.

Previous studies have found that CaSR expression is the strongest in lung cancer A549 cells with the highest bone metastasis ability. Based on the A549 cell line, a CaSR knockdown and overexpression cell line was constructed using a lentiviral expression system. Western blots confirmed CaSR expression levels after cell line construction. The results showed that protein expression in A549 cells with CaSR gene overexpression was significantly higher than that in the control group. CaSR protein expression in A549 cells was significantly lower after RNA interference than that in the negative control group (*p* < 0.05, Figure 2A).

### CaSR Enhances A549 Cell Proliferation and Migration

A549 cell proliferation was detected using CCK-8. A549 cell proliferation was higher in the CaSR overexpression group than in the control group and the difference was statistically significant (*p* < 0.001). However, A549 cell proliferation ability was significantly lower in the CaSR knockdown group than in the negative control group and the difference was statistically significant (*p* < 0.001, Figures 2B,D).

A549 cell invasion ability after overexpression and knockdown of CaSR gene was detected using a Transwell assay. The number of transmembrane cells among A549 cells overexpressing CaSR gene was significantly greater than that in the control group (Figures 2C,E). This difference was statistically significant (*p* < 0.001). The number of transmembrane cells was significantly lower in the CaSR knockdown group than that in the negative control group. This difference was statistically significant (*p* < 0.001). These results showed that A549 cell invasion ability was enhanced after CaSR gene overexpression and weakened after CaSR gene knockdown.

### CaSR Promotes Osteolytic Ability

**Figure 3:**
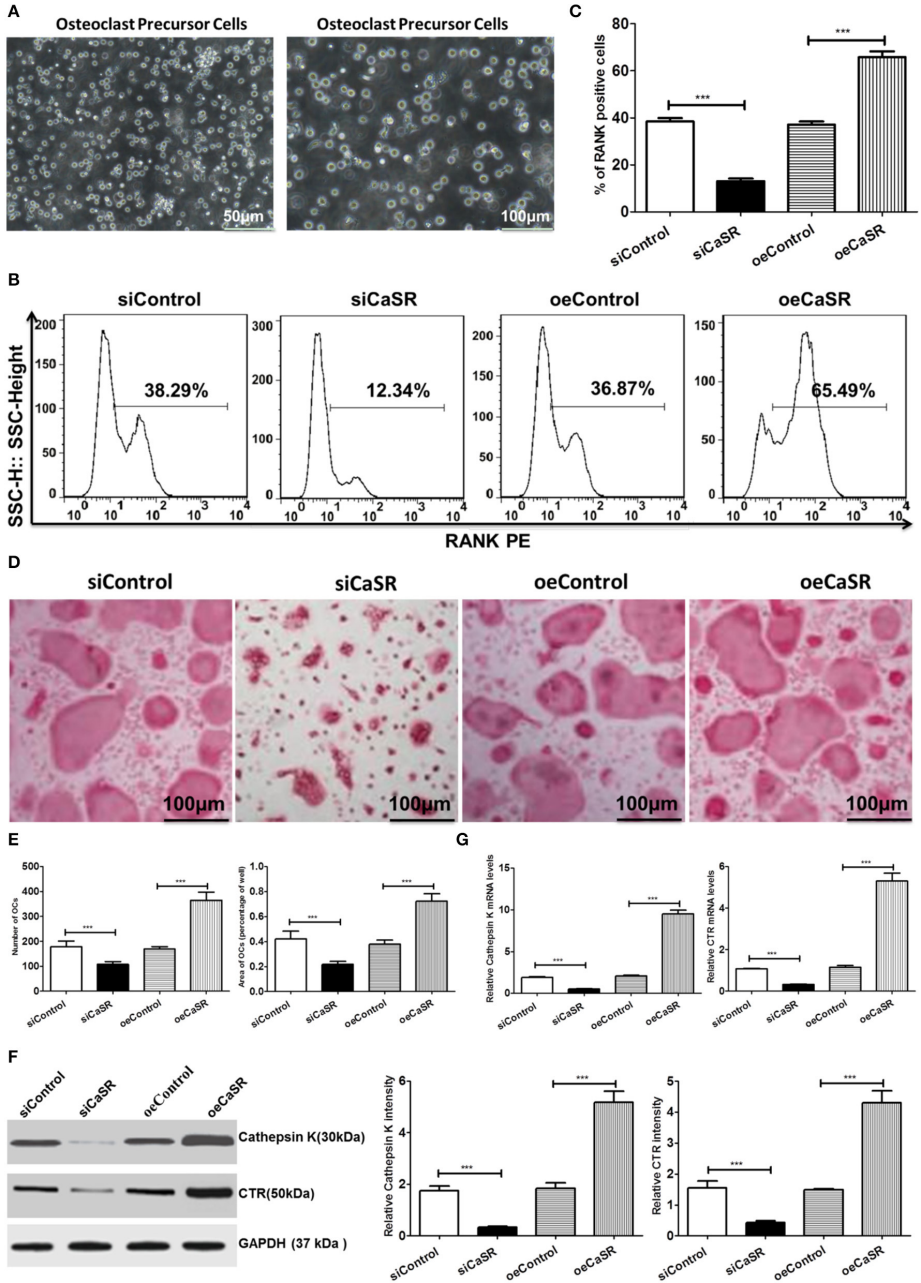
Analysis of CaSR effects on OCs. **(A)** Isolation and identification of OC precursor cells. **(B–E)** Four groups of OC precursor cells were treated with CM from modified A549 cells co-cultured with macrophages to examine potential OC differentiation induction. Flow cytometry was used to detect expression of RANK-positive OCs. TRAP+ multinucleated cells were counted using 400-fold microscope and TRAP-stained OC area was calculated using ImageJ software (NIH). OC number and osteolytic area size in CaSR overexpression group were significantly higher than those in empty vector and negative control groups (*p* < 0.001). The difference between negative and empty vector control groups was not statistically significant (*p* > 0.05). **(F)** Protein expression levels of bone matrix degradation-related enzymes cathepsin K and CTR were detected by western blot after CaSR overexpression and knockdown in A549 cells. ImageJ software (NIH) for image processing was used to verify band intensities as a result of semi-quantitative western blots. Protein expression levels of cathepsin K and CTR were significantly higher in CaSR overexpression group than in empty vector and negative control groups (*p* < 0.001). **(G)** The qPCR was used to detect expression of bone matrix degradation-related enzymes cathepsin K and CTR mRNA levels after CaSR overexpression and knockdown. Cathepsin K and CRT mRNA expression levels were significantly higher in CaSR overexpression group than in empty vector and negative control groups (*p* < 0.001). ***p < 0.001.

TRAP+ multinucleated cell count results showed that the number of OCs in the CaSR overexpression group was significantly higher than that in the empty vector and negative control groups. There was no statistically significant difference between the empty vector and negative control groups. The representative marker RANK was stained for OCs and demonstrated that percentage of RANK-positive cells in the CaSR knockdown group was much lower than that in the empty vector and negative control groups (*p* < 0.001). The percentage of RANK-positive cells in the CaSR overexpression group was significantly higher than that in the empty vector and negative control groups (*p* < 0.001, Figures 3B,C). The percentage of osteolytic area in the total field of view was then calculated. The results showed that the osteolytic area in the CaSR overexpression group was significantly higher than that in the empty vector and negative control groups (*p* < 0.001). The osteolytic area in the negative control group was slightly higher than that in the empty vector control group, but the difference was not statistically significant (*p* > 0.05). The increase in the number and osteolytic area in the CaSR overexpression group suggested that CaSR promoted the osteolytic function of OCs and significantly enhanced their osteolytic ability (Figures 3D,E). OC number and osteolytic area size in CaSR overexpression A549 cells plus macrophages group (oeCaSR + MΦ) were significantly higher than those in CaSR knock-down A549 cells plus macrophages group (siCaSR + MΦ), the CaSR-modulated A549 cells alone groups (siCaSR and oeCaSR), and the macrophages alone group (MΦ) (*p* < 0.001). The difference between the CaSR-modulated A549 cells alone groups (siCaSR and oeCaSR), and the macrophages alone group (MΦ) were not statistically significant (*p* > 0.05) (see Figure S1).

Cathepsin K and CTR are secreted by OCs and are related to bone matrix degradation. Their expression levels were verified in the study. The qPCR and western blot were used to detect the expression of bone matrix degradation-related enzymes cathepsin K and CTR mRNA and protein after CaSR overexpression or knockdown. Cathepsin K, CTR mRNA, and protein expression levels were significantly higher in the CaSR overexpression group than in the empty vector and negative control groups (*p* < 0.001) (Figures 3F,G).

**Figure 4:**
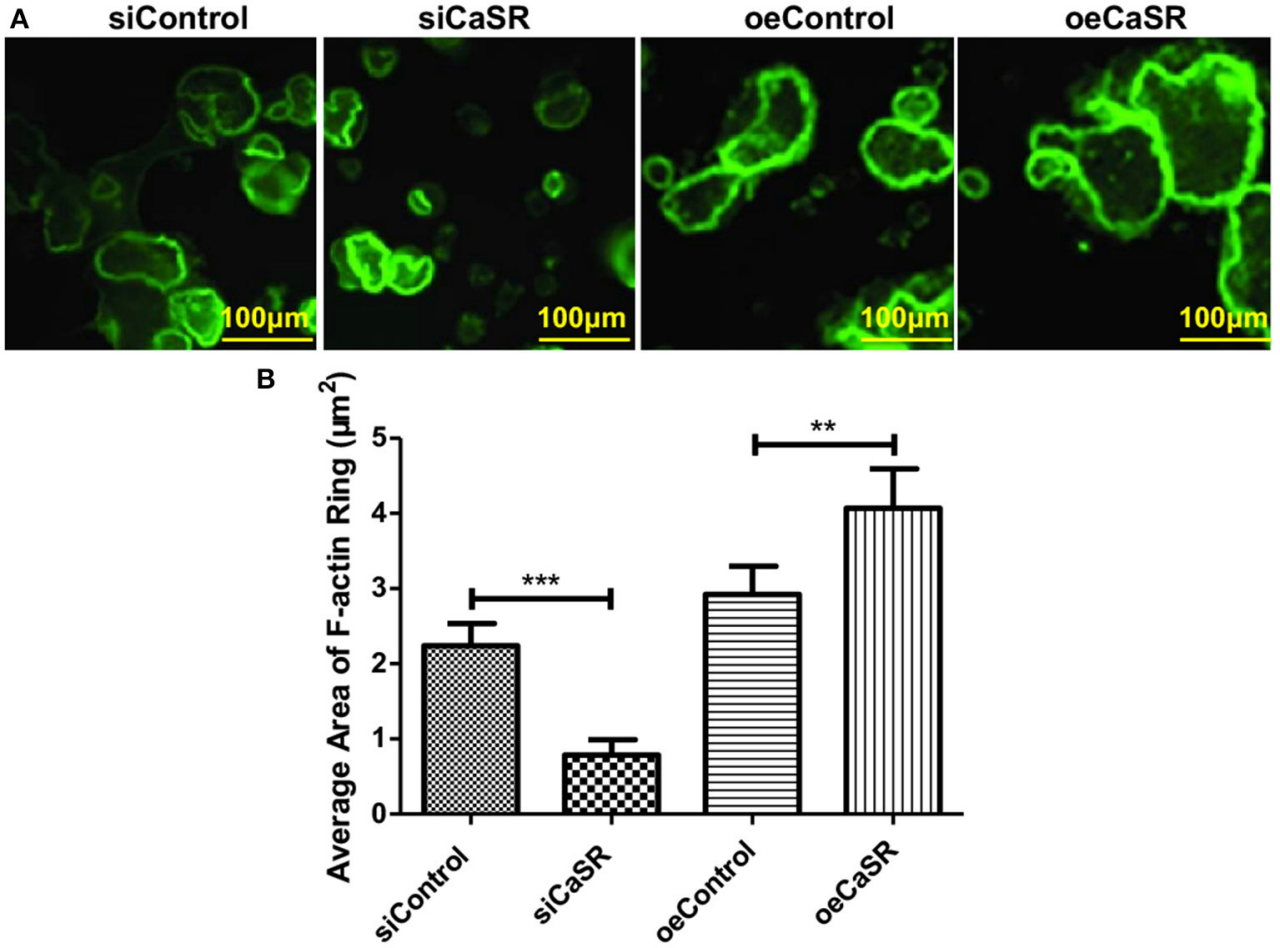
CaSR effect on F-actin ring formation of OCs. **(A,B)** Four groups of OC precursor cells were treated with CM from modified A549 cells co-cultured with macrophages to examine potential OC differentiation induction. Immunofluorescent staining was used to evaluate F-actin ring formation of OCs. The average area of F-actin ring of OCs was detected and calculated using 400-fold microscope and ImageJ software (NIH). The average area of F-actin ring of OCs in CaSR overexpression group was significantly higher than that in empty vector and negative control groups (*p* < 0.001). The difference between negative and empty vector control groups was not statistically significant (*p* > 0.05). **p < 0.01, ***p < 0.001.

The formation of F-actin rings is a prerequisite to the adhesion of osteoclasts to bone. We evaluate F-actin ring formation using immunofluorescent staining. The average area of F-actin ring of OCs in CaSR overexpression group was significantly higher than that in empty vector and negative control groups (*p* < 0.001). The difference between negative and empty vector control groups was not statistically significant (*p* > 0.05) (Figures 4A,B).

### CaSR Reduces the Number of OBs and Osteogenic Area

**Figure 5:**
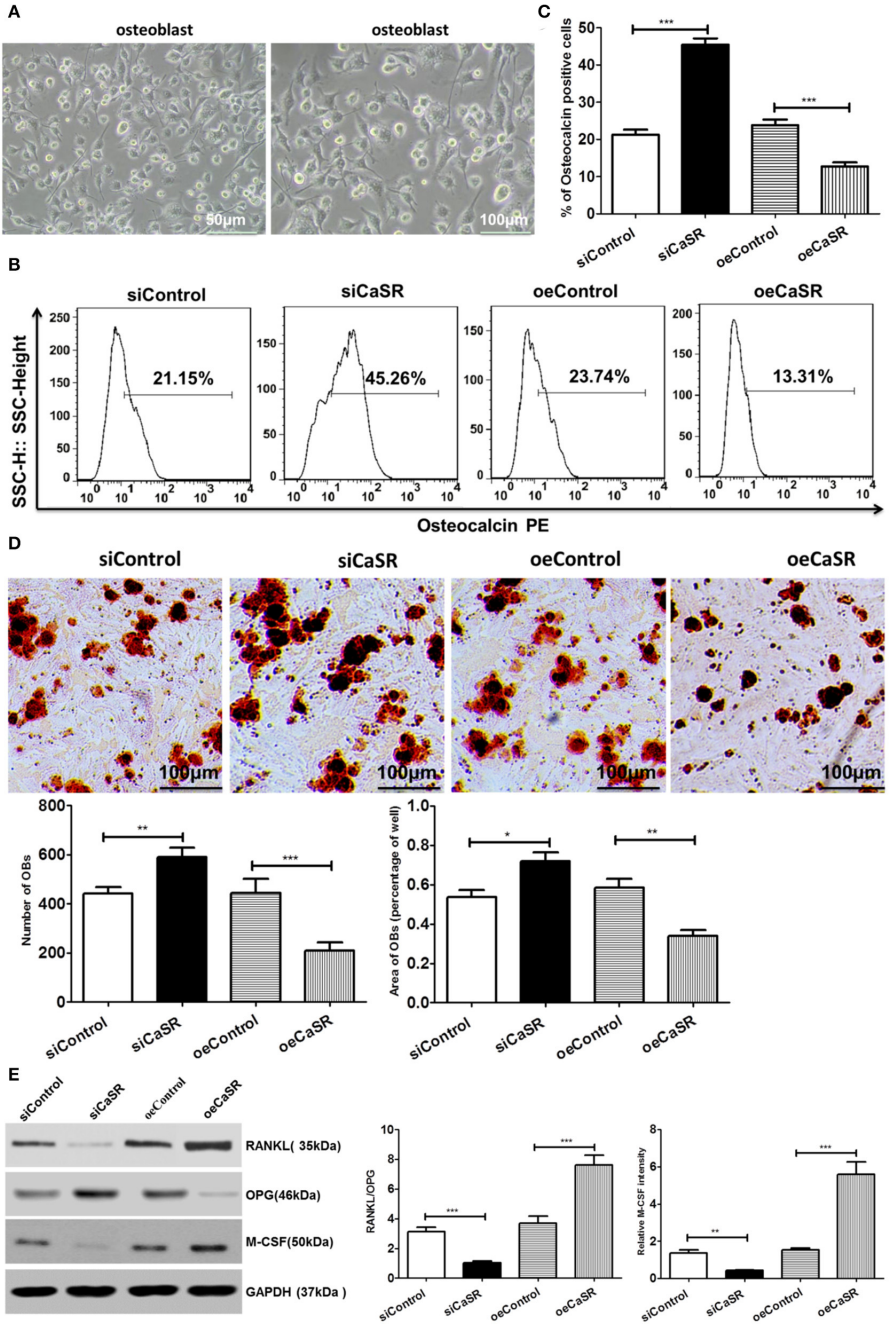
Analysis of CaSR effects on OBs. **(A)** Primary culture and identification of rat OBs. **(B–D)** Four groups of OB precursor cells were treated with CM from co-cultured modified A549 cells with macrophages to examine potential OB differentiation induction. Flow cytometry was used to detect expression of osteocalcin-positive OBs. OB mineralization nodule staining was performed with Alizarin Red S (AS). AS+ cells were counted using 400-fold microscope and AS-stained OB area was calculated using ImageJ software (NIH). The number of OBs in CaSR overexpression group was significantly lower than that in empty vector and negative control groups (*p* < 0.001). There was no significant difference between empty vector and negative control groups (*p* > 0.05). Osteogenic area in CaSR overexpression group was significantly smaller than that in empty vector and negative control groups (*p* < 0.001). While there was a difference between negative and empty vector control groups, this difference was not statistically significant (*p* > 0.05). OB number and osteogenic area size were significantly higher in CaSR knockdown group than in empty vector and negative control groups (*p* < 0.001). **(E)** OB expression induced OC maturation and differentiation enzymes RANKL, M-CSF, and OPG were detected using western blots. ImageJ software (NIH) for image processing was used to verify band intensities as a result of semi-quantitative western blotting. RANKL and M-CSF protein expression was significantly higher in CaSR overexpression group than in empty vector control group. OPG protein expression level was significantly lower in CaSR overexpression group than in empty vector and negative control groups (*p* < 0.001). *p < 0.05, **p < 0.01, ***p < 0.001.

The primary culture and identification results of rat OBs are shown in Figure 5A. The number of OBs in the CaSR overexpression group was significantly lower than that in the empty vector and negative control groups (*p* < 0.001). There was no significant difference between the empty vector and negative control groups (*p* > 0.05). The representative marker osteocalcin was stained for OBs and revealed that the number of OBs in the CaSR overexpression group was significantly lower than that in the empty vector and negative control groups (*p* < 0.001). There was no significant difference between the empty vector and negative control groups (*p* > 0.05) (Figures 5B,C). Osteogenic area in the CaSR overexpression group was significantly lower than that in the empty vector and negative control groups (*p* < 0.001). Osteogenic area in the negative control group was slightly higher than that in the empty vector control group, but the difference was not statistically significant (*p* > 0.05). The number of OBs and size of osteogenic area were significantly higher in the CaSR knockdown group than in the empty vector and negative control groups (*p* < 0.001) (Figure 5D). Western blot was used to detect the expression of OB-induced OC differentiation-related enzymes RANKL, M-CSF, and osteoprotegerin (OPG). RANKL is the most accurate predictor of bone response in patients with bone metastases and is the target of novel monoclonal antibody denosumab (19). M-CSF serves as a cofactor in RANKL-stimulated generation of OCs from hematopoietic precursors (20, 21). RANKL and M-CSF expression levels were significantly higher in the CaSR overexpression group than in the empty vector and negative control groups. OPG expression level was significantly lower than that in the empty vector and negative control groups (*p* < 0.001) (Figure 5E). The ratio of RANKL/OPG was significantly increased in the CaSR overexpression group. Therefore, RANKL and OPG secretion could regulate local osteolytic balance and induce OC differentiation.

**Figure 6:**
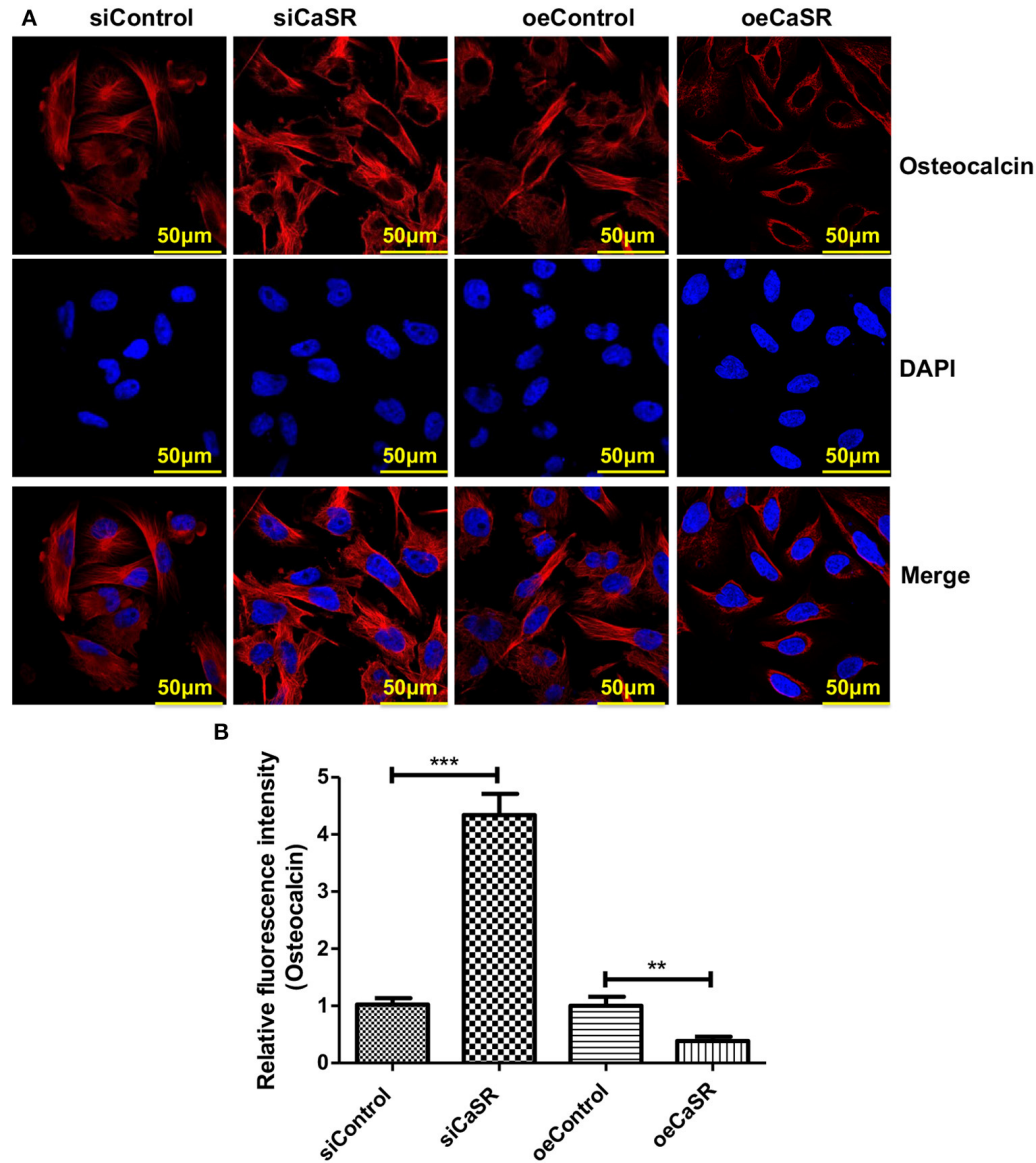
CaSR effect on osteocalcin expression of OBs. **(A,B)** Four groups of OB precursor cells were treated with CM from co-cultured modified A549 cells with macrophages to examine potential OB differentiation induction. Immunofluorescent staining was used to detect expression of osteocalcin-positive OBs. The relative fluorescence intensity of osteocalcin of OBs in CaSR overexpression group was significantly lower than that in empty vector and negative control groups (*p* < 0.001). There was no significant difference between empty vector and negative control groups (*p* > 0.05). **p < 0.01, ***p < 0.001.

Immunofluorescent staining was also used to detect expression of the representative marker osteocalcin of OBs. The relative fluorescence intensity of osteocalcin of OBs in CaSR overexpression group was significantly lower than that in empty vector and negative control groups (*p* < 0.001). There was no significant difference between empty vector and negative control groups (*p* > 0.05) (Figures 6A,B).

### CaSR Is Involved in Bone Metastasis via Activation of NF-κB in Lung Adenocarcinoma

**Figure 7:**
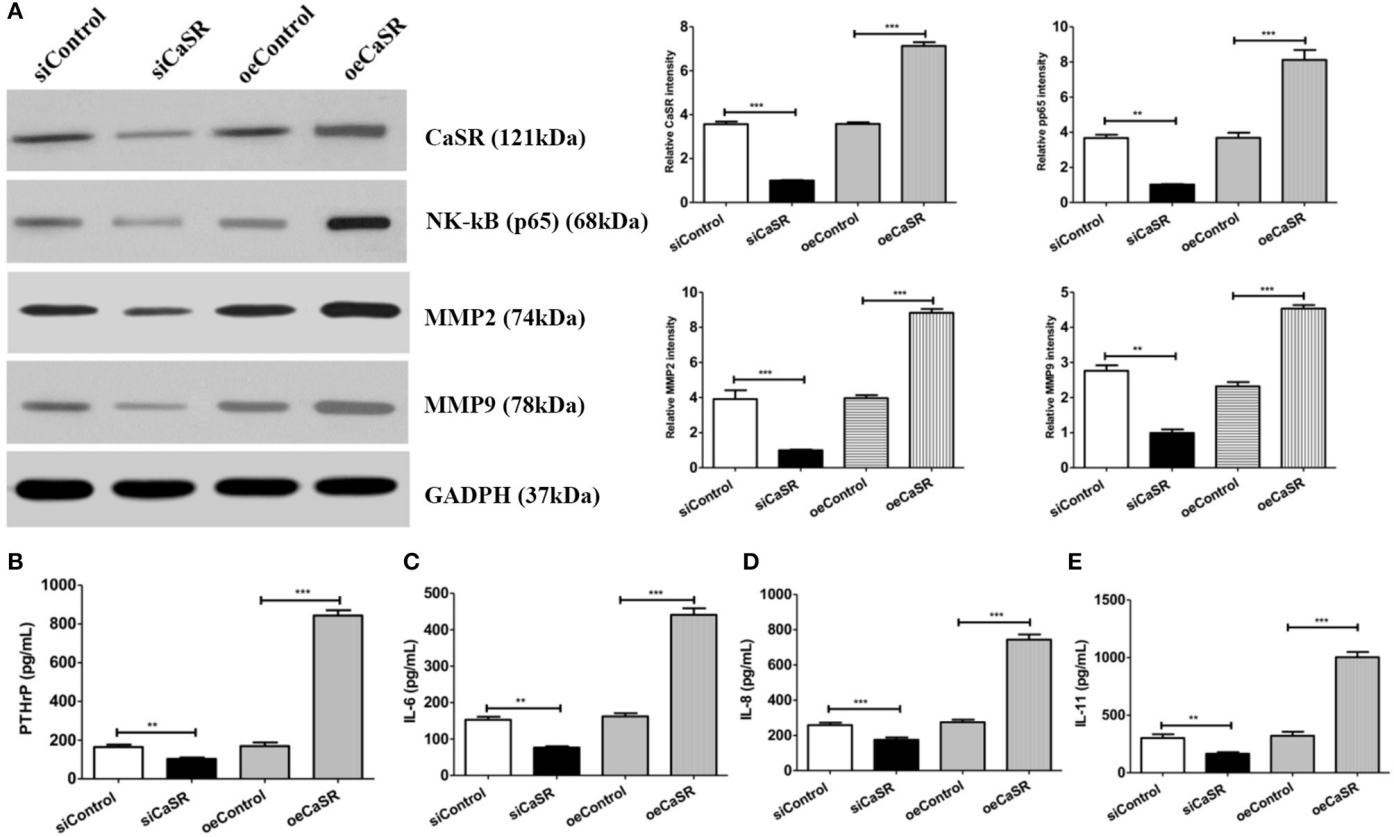
Analysis of cytokine expression levels. **(A)** Western blotting confirmed NF-κB, MMP2, and MMP9 expression levels. ImageJ software (NIH) was used for image processing to verify band intensities as a result of semi-quantitative western blots. NF-κB expression level in CaSR overexpression group was significantly higher than that in empty vector and negative control groups. NF-κB, MMP2, and MMP9 expression levels were significantly lower in CaSR knockdown group than in empty vector and negative control groups (*p* < 0.001). **(B)** PTHrP expression levels in CM from modified A549 cells co-cultured with macrophages were detected by ELISA. PTHrP expression level was significantly higher in CaSR overexpression group than in empty vector and negative control groups. PTHrP expression level was significantly lower in CaSR knockdown group than in empty vector and negative control groups (*p* < 0.001). **(C–E)** IL-6, IL-8, and IL-11 expression in CM from modified A549 cells co-cultured with macrophages were detected by ELISA. IL-6, IL-8, and IL-11 expression levels were significantly higher in CaSR overexpression group than in empty vector and negative control groups. Expression levels of these three factors were significantly lower in CaSR knockdown group than in empty vector and negative control groups (*p* < 0.001). **p < 0.01, ***p < 0.001.

Western blot was used to confirm the effect of CaSR on NF-κβ protein expression in order to further analyze the mechanism of CaSR in bone metastasis of lung adenocarcinoma. NF-κβ protein expression increased when CaSR was overexpressed and decreased when CaSR was silenced. The results showed that NF-κβ expression in the CaSR overexpression group was significantly higher than that in the empty vector and negative control groups. NF-κβ expression in the CaSR silenced group was much lower than that in the empty vector and negative control groups (*p* < 0.001). The results indicated that CaSR expression level affected the expression of NF-κβ. As expected, MMP2 and MMP9 expression was increased after CaSR overexpression and the invasion ability was enhanced. MMP2 and MMP9 expression was decreased after CaSR was knocked down and the invasion ability was weakened, which was consistent with the observed phenotype (Figure 7A).

CaSR can promote PTHrP secretion when it is upregulated in the tumor cell. In the present experiment, PTHrP expression after CaSR overexpression was detected using ELISA. PTHrP expression was significantly higher in the CaSR overexpression group than in the empty vector and negative control groups. PTHrP expression was significantly lower in the CaSR knockdown group than in the empty vector and negative control groups (*p* < 0.001) (Figure 7B). PTHrP can directly induce OC differentiation and maturation by promoting secretion of IL-6, IL-8, IL-11, and other OC differentiation factors. The experiment further examined the expression of IL-6, IL-8, and IL-11. IL-6, IL-8, and IL-11 expression levels were significantly higher in the CaSR overexpression group than in the empty vector and negative control groups. The expression levels of the three factors were significantly lower in the CaSR knockdown group than in the empty vector and negative control groups (*p* < 0.001) (Figures 7C–E).

## Discussion

Tumor recurrence and metastasis are the main causes of high mortality in lung adenocarcinoma (22). Lung adenocarcinoma is highly invasive and fatal. Approximately 65% of patients have bone metastases at the time of diagnosis. Most of them have osteolytic destruction and a few have osteogenic destruction (23). Bone metastasis location is often the site of bone metabolism. Calcium ions that are widely present in bone microenvironment can be used as a chemokine. Tumor cells with a high expression of CaSR are recruited to reach local bone tissue via EMT and directly or indirectly act on OCs in the bone microenvironment, which disrupts the balance of bone metabolism, promotes OC differentiation and maturation, and forms new bone metastases (6, 7, 24). Studies have shown that tumor cells with a high expression of CaSR are more susceptible to bone metastasis than tumor cells with a low expression of CaSR (6, 7).

Clinical studies have confirmed that normal lung tissue has a low or negative CaSR expression (9). This study first analyzed 120 samples from lung adenocarcinoma patients and found that CaSR expression in lung cancer was significantly higher compared to normal lung and adjacent tissues. Moreover, lung adenocarcinoma patients with bone metastasis had higher levels of CaSR expression in lung cancer tissues compared to patients without bone metastasis (Figure 1). In addition, our previous study also found that stronger bone metastatic ability in the lung adenocarcinoma cell line resulted in a higher expression of CaSR. These results point to a process by which CaSR may be involved in bone metastasis of lung adenocarcinoma.

To test this hypothesis, the present study constructed stable CaSR overexpression and knockdown transfected cell lines with a high metastatic potential using A549 cells (Figure 2A). A549 cell proliferation and invasion abilities were significantly increased when CaSR was overexpressed (Figures 2B,C). Moreover, increased expression levels of MMP2 and MMP9 helped to enhance tumor cell invasion ability (Figure 7A). A549 cell proliferation and invasion abilities were significantly decreased when CaSR expression was knocked down. These CaSR biological behaviors affecting tumor cells suggest that CaSR is involved in the process of lung adenocarcinoma development and bone metastasis.

Studies have revealed that tumor cells and macrophages can establish a symbiotic relationship in the tumor microenvironment, where macrophages generate an inflammatory microenvironment in favor of cancer growth (4). Cross-talk with the inflammatory microenvironment may alter tumor cell properties, which may disrupt bone-forming and bone-resorbing activities. Eventually, it can lead to macrometastasis in the bone. Chemokines play an important role in mediating the crosstalk between tumor cells and bone microenvironment (16). Some chemokines play critical roles in bone destruction derived from tumor cells (16). Therefore, osteolytic and osteogenic changes in response to CaSR overexpression and knockdown were analyzed using CM from modified A549 cells co-cultured with macrophages. CM obtained from the co-culture system directly stimulated OC precursor cells. Cell counts showed that after CaSR overexpression in the A549 cells, the number of OCs and the size of osteolytic area, F-actin ring formation increased significantly and expression levels of bone matrix degradation-related enzymes cathepsin-K and CTR increased significantly on the mRNA and protein levels. The number of OBs, the expression of osteocalcin and the size of osteolytic area significantly decreased and expression of OB-induced OC maturation and differentiation enzymes RANKL/OPG and M-CSF was also significantly elevated. These results were reversed after CaSR expression was knocked down in the A549 cells (Figures 3A, 5A). Increased bone matrix degrading enzymes led to more bone matrix degradation. RANKL and OPG secretion levels can regulate local osteolytic balance, induce OC differentiation, and disrupt the dynamic balance of bone tissue microenvironment. OC differentiation and maturation and initiation of osteolysis is the starting point of bone metastasis in lung adenocarcinoma (25).

After lung adenocarcinoma A549 cells were co-cultured with macrophages, NF-κB was activated in lung adenocarcinoma A549 cells and tumor cell proliferation and invasion abilities were significantly enhanced. The observed phenotype was consistent with previous research (26, 27). The CaSR promoter region, which contains a functional response element κB, upregulates the NF-κB signaling pathway, activates CaSR, and participates in pathological processes, such as inflammatory bone resorption (28–30). In this study, NF-κB expression after CaSR overexpression and knockdown was examined. It was found that the expression of NF-κB increased when CaSR was overexpressed. The result was reversed when CaSR was knocked down. It indicated that CaSR may positively regulate NF-kB.

PTHrP is a key OC differentiation factor produced by tumor cells that is essential for the induction of OC maturation and differentiation. It has been shown that PTHrP expression is significantly increased in cancer tissues with bone metastases and is positively correlated with bone metastasis of lung cancer (13). PTHrP is mainly regulated by its upstream gene CaS (11). When CasR on the surface of tumor cells is upregulated and activated, it can stimulate PTHrP secretion from tumor cells (6). PTHrP can induce RANKL-RANK binding by inducing OBs to express RANKL. It can disrupt bone metabolism balance by inhibiting OPG secretion. PTHrP can also directly induce OC differentiation and maturation by promoting secretion of IL-8, IL-6, IL-11, and other OC differentiation factors by tumor cells (Figures 7C–E). The results confirmed that secretion of PTHrP was regulated by CaSR in the culture supernatant of each group of cells and varied based on CaSR expression (Figure 7B). As a downstream signaling molecule of CaSR, PTHrP plays an important role in bone metastasis of lung adenocarcinoma (10).

Our preliminary study demonstrated that in A549 cells with a high metastatic potential, CaSR can induce RANKL expression of OBs and inhibit OPG expression, thus disrupting the balance of bone metabolism. CaSR can also promote the differentiation and maturation of OCs. The underlying mechanisms may be partly due to the activation of CaSR which can promote NF-κB expression and enhance PTHrP release, thus eventually promoting the development of bone metastasis in lung adenocarcinoma.

## Data Availability Statement

The datasets generated for this study are available on request to the corresponding author.

## Ethics Statement

The studies involving human participants were reviewed and approved by the medical ethics review board of Beijing Friendship Hospital, Capital Medical University. The participants provided their written informed consent to participate in this study.

### Standard Biosecurity and Institutional Safety Procedures

All procedures performed in studies were in accordance with the standard biosecurity and institutional safety procedures of the institutional research committee.

## Author Contributions

JY had substantial contributions to the conception and design of the work. LL and YF wrote all sections of the manuscript. ZC and YZ provided the literature and revised this article.

### Conflict of Interest

The authors declare that the research was conducted in the absence of any commercial or financial relationships that could be construed as a potential conflict of interest.

## Supplementary Material

Figure S1 CM effect on OC differentiation induction. Five groups of OC precursor cells were treated with CM from the CaSR-modified A549 cells plus macrophages, the CaSR-modulated A549 cells alone, and the macrophages alone groups to examine potential OC differentiation induction. TRAP+ multinucleated cells were counted using 400-fold microscope and TRAP-stained OC area was calculated using ImageJ software (NIH). OC number and osteolytic area size in CaSR overexpression A549 cells plus macrophages group (oeCaSR + MΦ) were significantly higher than those in CaSR knock-down A549 cells plus macrophages group (siCaSR + MΦ), the CaSR-modulated A549 cells alone groups (siCaSR and oeCaSR), and the macrophages alone group (MΦ) (*p* < 0.001). The difference between the CaSR-modulated A549 cells alone groups (siCaSR and oeCaSR), and the macrophages alone group (MΦ) were not statistically significant (*p* > 0.05). **(A)** CaSR knock-down A549 cells plus macrophages group (siCaSR + M_Φ_). **(B)** CaSR overexpression A549 cells plus macrophages group (oeCaSR + M_Φ_). **(C)** CaSR knock-down A549 cells alone (siCaSR). **(D)** CaSR overexpression A549 cells alone (oeCaSR). **(E)** macrophages alone (M_Φ_). ^***^*p* < 0.001.
