# Supplementary figures and images for: CaSR Induces Osteoclast Differentiation and Promotes Bone Metastasis in Lung Adenocarcinoma
Source: Front Oncol. 2020 Mar 25;10:305. doi: 10.3389/fonc.2020.00305 (PMC7109411; doi:10.3389/fonc.2020.00305)

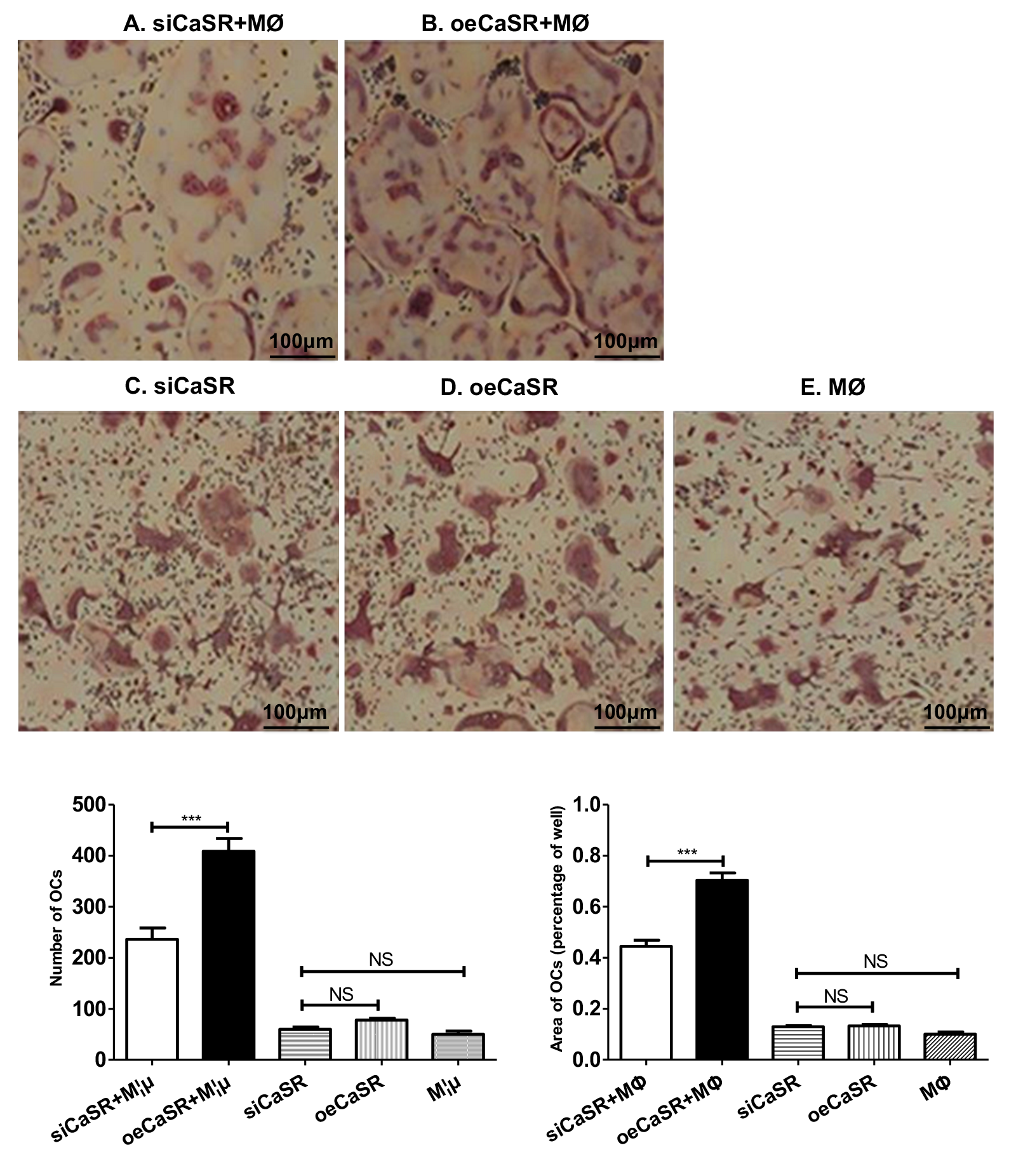

Supplement: Figure S1 — CM effect on OC differentiation induction. Five groups of OC precursor cells were treated with CM from the CaSR-modified A549 cells plus macrophages, the CaSR-modulated A549 cells alone, and the macrophages alone groups to examine potential OC differentiation induction. TRAP+ multinucleated cells were counted using 400-fold microscope and TRAP-stained OC area was calculated using ImageJ software (NIH). OC number and osteolytic area size in CaSR overexpression A549 cells plus macrophages group (oeCaSR + MΦ) were significantly higher than those in CaSR knock-down A549 cells plus macrophages group (siCaSR + MΦ), the CaSR-modulated A549 cells alone groups (siCaSR and oeCaSR), and the macrophages alone group (MΦ) (p < 0.001). The difference between the CaSR-modulated A549 cells alone groups (siCaSR and oeCaSR), and the macrophages alone group (MΦ) were not statistically significant (p > 0.05). (A) CaSR knock-down A549 cells plus macrophages group (siCaSR + MΦ). (B) CaSR overexpression A549 cells plus macrophages group (oeCaSR + MΦ). (C) CaSR knock-down A549 cells alone (siCaSR). (D) CaSR overexpression A549 cells alone (oeCaSR). (E) macrophages alone (MΦ). ***p < 0.001. [file Image_1.TIF]
